# Inflammatory pre-conditioning restricts the seeded induction of α-synuclein pathology in wild type mice

**DOI:** 10.1186/s13024-016-0142-z

**Published:** 2017-01-03

**Authors:** Emily J. Koller, Mieu M. T. Brooks, Todd E. Golde, Benoit I. Giasson, Paramita Chakrabarty

**Affiliations:** 1 Department of Neuroscience, University of Florida, Gainesville, Florida 32610 USA; 2 Center for Translational Research in Neurodegenerative Disease, University of Florida, Gainesville, Florida 32610 USA; 3 McKnight Brain Institute, University of Florida, 1275 Center Drive, PO Box 100159, Gainesville, Florida 32610 USA; 4 Present Address: Department of Neuroscience, Mayo Clinic, Jacksonville, FL 32224 USA

**Keywords:** α-synuclein, Gliosis, IL-6, Inclusion pathology, Seeding

## Abstract

**Background:**

Cell-to-cell transmission of α-synuclein (αSyn) is hypothesized to play an important role in disease progression in synucleinopathies. This process involves cellular uptake of extracellular amyloidogenic αSyn seeds followed by seeding of endogenous αSyn. Though it is well known that αSyn is an immunogenic protein that can interact with immune receptors, the role of innate immunity in regulating induction of αSyn pathology in vivo is unknown. Herein, we explored whether altering innate immune activation affects induction of αSyn pathology in wild type mice.

**Methods:**

We have previously demonstrated that recombinant adeno-associated virus (AAV) mediated expression of the inflammatory cytokine, Interleukin (IL)-6, in neonatal wild type mice brains leads to widespread immune activation in the brain without overt neurodegeneration. To investigate how IL-6 expression affects induction of αSyn pathology, we injected mouse wild type αSyn fibrils in the hippocampus of AAV-IL-6 expressing mice. Control mice received AAV containing an Empty vector (EV) construct. Two separate cohorts of AAV-IL-6 and AAV-EV mice were analyzed in this study: 4 months or 2 months following intrahippocampal αSyn seeding.

**Results:**

Here, we show that IL-6 expression resulted in widespread gliosis and concurrently reduced αSyn inclusion pathology induced by a single intra-hippocampal injection of exogenous amyloidogenic αSyn. The reduction in αSyn inclusion pathology in IL-6 expressing mice was time-dependent. Suppression of αSyn pathology was accompanied by reductions in both argyrophilic and p62 immunoreactive inclusions.

**Conclusions:**

Our data supports a beneficial role of inflammatory priming of the CNS in wild type mice challenged with exogenous αSyn. A likely mechanism is efficient astroglial scavenging of exogenous αSyn, at least early in the disease process, and in the absence of human αSyn transgene overexpression. Given evidence that a pro-inflammatory environment may restrict seeding of αSyn pathology, this can be used to design anti-αSyn immunobiotherapies by harnessing innate immune function.

**Electronic supplementary material:**

The online version of this article (doi:10.1186/s13024-016-0142-z) contains supplementary material, which is available to authorized users.

## Background

Synucleinopathies are a group of neurodegenerative diseases characterized by the presence of intra-cytoplasmic amyloidogenic inclusions comprised of the protein α-synuclein (αSyn) [1, 2]. In most of these disorders, such as Parkinson’s disease (PD) or dementia with Lewy bodies (DLB), these αSyn inclusions are predominantly neuronal [2, 3]. The majority of pathological αSyn in these inclusions are phosphorylated at the serine 129 residue and this modification is generally used as a biological marker to monitor inclusion formation when combined with other histological assays [3, 4].

The aberrant aggregation of αSyn to form amyloidogenic inclusions is thought to follow a prion-like mechanism involving the molecular conversion of protein monomers from their predominantly unfolded structure to a β-pleated sheet that can then polymerize into amyloid (reviewed in [4]). As part of this prionoid mechanism, such conformationally altered forms of αSyn can readily aggregate and can subsequently be propagated between cells [5–12]. Consistent with this model, both intracerebral or peripheral injections of recombinant αSyn amyloid seeds result in robust induction of αSyn pathology in αSyn transgenic mice and to a lesser extent, in wild type mice [6, 7, 9, 13–15]. Modeling studies have further shown that both soluble and aggregated αSyn can be released and taken up by cells via various mechanisms [16–18], providing proof of concept that pathological αSyn shares prion-type transmission properties.

The observations that innate immune activation is an invariant finding in synucleinopathies and that αSyn, by itself, can directly interact with immune cells suggest that innate immunity can potentially modify how exogenous αSyn is able to influence the onset and progression of α-synucleinopathy (reviewed in [19, 20]). In mouse models of αSyn inclusion pathology induced by exogenous αSyn aggregates, a significant component of the αSyn pathology is retained in glial cells [7, 21]. Glial cytoplasmic inclusions (Papp-Lantos bodies) and tuft-shaped astrocytes laden with aggregated αSyn have been reported in patients with synucleinopathies, such as PD, multiple system atrophy (MSA) and DLB [22–25] as well as in transgenic mouse models of α-synucleinopathy [26–28]. Mechanistically, extracellular αSyn can directly activate microglia by interacting with microglial receptors including TLR2 and TLR4 [29–31]. CNS resident astrocytes as well as macrophages can endocytose αSyn via dynamin-related pathways [16, 17], suggesting that immune pathways can potentially have disease modifying effects in α-synucleinopathies.

To investigate how innate immune activation alters seeded α-synucleinopathy in wild type mice, we used an Interleukin (IL)-6 driven somatic transgenesis model. IL-6 is a pleiotropic cytokine that plays a key role in immune regulation, hematopoiesis and acute phase reactions [32]. Under chronic conditions, IL-6 induces an acute inflammatory condition by activating immune cells, such as macrophages, B cells, microglia and astrocytes. Using recombinant adeno-associated viruses overexpressing IL-6 in the brains of wild type mice [33], we explored how preconditioning innate immune milieu in the CNS affects the induction of αSyn pathology following challenge with exogenous aggregated αSyn. IL-6 expression, as expected, leads to widespread astrogliosis and, surprisingly, attenuates the induction of αSyn pathology in these mice. Thus, contrary to our expectations that an inflammatory milieu might exacerbate α-synucleinopathy, we report that IL-6 induced immune preconditioning limits induction of α-synucleinopathy following injection of exogenous αSyn aggregates.

## Methods

### Mice

All mouse husbandry and experimental procedures were conducted in accordance with IACUC and University of Florida policies. All mice were maintained under a 12 h light/dark cycle and had access to food and water ad libitum.

### Preparation of recombinant proteins and αSyn fibrils

Recombinant full length mouse wild type αSyn was purified and fibrillized as described before [13, 34]. Briefly, recombinant αSyn was expressed and purified from *E. coli*, assembled in vitro into fibrils in PBS buffer and gently fragmented for 60 min using a bath sonicator before injection. Presence of endotoxins was assessed using the TLR4 assay (InvivoGen). The experiments were done using batches of fibrillar αSyn seeds validated by K114 fluorescence and their ability to induce αSyn pathology in HEK293 cells [35]. Following validation steps, these fibrils were aliquoted and stored at −80 °C till use.

### rAAV delivery and hippocampal stereotactic injections

rAAV were prepared as described previously [33]. Wild type B6/C3H mice received bilateral, intraventricular injections of rAAV (capsid 1) expressing the inflammatory cytokine IL-6 or the empty vector plasmid containing no expression cassette (EV) on neonatal day P0 as described previously [33]. Mice were aged to 2 months and bilaterally injected (coordinates from Bregma: A/P −2.2, L +/−1.6, D/V −1.2) with pre-fibrillized wild type mouse αSyn aggregates in the hippocampus according to Sacino et al [7]. Fibrils (2 μL of 1mg/ml synuclein fibrils per hemisphere) were injected at a rate of 0.2 μL per min. Control mice were injected with sterile PBS in the hippocampus. Mice used in various cohorts are shown in Table 1.

**Table 1:** Mice used in study

rAAV1 injection (P0)	Hippocampal injection (2 mo)	Harvest age	*n*	Used in
Naive	N/A	1.5 mo	4	Fig. 1, Additional file 1: Figure S1
rAAV1-IL-6	N/A	1.5 mo	4	Fig. 1, Additional file 1: Figure S1
rAAV1-EV	αSyn	6 mo	4	Figs. 2, 3, 4 and 5, Additional file 2: Figure S2 and Additional file 3: Figure S3
rAAV1-EV	PBS	6 mo	5	Figs. 2, 3, 4 and 5, Additional file 2: Figure S2, Additional file 3: Figure S3 and Additional file 4: Figure S4
rAAV1-IL6	αSyn	6 mo	5	Figs. 2, 3, 4 and 5, Additional file 2: Figure S2 and Additional file 3: Figure S3
rAAV1-IL6	PBS	6 mo	3	Figs. 2, 3, 4 and 5, Additional file 2: Figure S2, Additional file 3: Figure S3 and Additional file 4: Figure S4
rAAV1-EV	αSyn	4 mo	5	Fig. 6, Additional file 5: Figure S5
rAAV1-IL6	αSyn	4 mo	5	Fig. 6, Additional file 5: Figure S5

### Immunohistochemistry

Mice were euthanized by intra-cardiac perfusion and brains were fixed in neutral buffered formalin. Paraffin-embedded brain sections were assessed with the following antibodies: GFAP (Cell Signaling, 1:1000), Iba1 (Wako, 1:1000); pSer129/81A αSyn (1:3000; [13, 36]); EP1536Y (AbCam, 1:1000); p62 (Protein Tech; 1:1000), cd11b (AbCam; 1:250), MHCII (Novus; 1:50) and NeuN (Abcam; 1:500). For all antibodies except cd11b and MHCII, antigen retrieval was performed by steaming for 25 min in water. For cd11b and MHCII, antigen retrieval was done by steaming in Dako Target Retrieval Solution S1699 (modified citrate buffer, pH 6.1). Colorimetric slides were treated with ImmPress reagents (Vector Labs) and visualized with 3, 3’diaminobenzidine followed by hematoxylin counterstaining. Fluorimetric slides were visualized with Alexa Fluor conjugated secondary antibodies (Invitrogen) and counterstaining with 4’, 6-diamidino-2-phenylindole (DAPI; Southern Biotech). All colorimetric slides were scanned using the Aperio XT whole slide scanner while fluorescent slides were visualized using Olympus BX60 microscope with a color digital camera.

### Modified Campbell-Switzer silver staining

This was performed based on the original protocol by Switzer [37]. Briefly, brain sections were placed in 2% ammonium hydroxide for 15 min followed by incubation in Silver-Pyridine-Carbonate solution in the dark with gentle stirring for 40 min. Slides were treated consecutively in 1% citric acid and acetate buffer (pH 5.0) followed by immersion in the developer solution (sodium carbonate, ammonium nitrate, silver nitrate and formalin). Color development was stopped by transferring slides to acetate buffer and further incubation in sodium thiosulfate for 30 s. Finally, slides were dehydrated in a series of alcohols and coverslips were applied.

### Quantification of gliosis and spatial depiction of pathology in brain maps

Paraffin-embedded brain sections were stained with anti GFAP and anti Iba-1 antibodies, images captured by Aperio XT scanner and analyzed using the Positive Pixel Count algorithm in ImageScope (Aperio). At least three slides were used to calculate an averaged immunostaining burden quantity by a blinded experimenter.

CNS distribution of αSyn pathology was performed by a blinded experimenter by visually evaluating slides from each experiment (4 month or 2 month cohorts) in a single sitting on an Evos light microscope (ThermoFisher). A secondary reviewer also performed random spot-checks to establish colorimetric assessment. Presence of αSyn pathology is represented by red dots on a brain map to reflect the relative levels of pathology in a qualitative manner. To quantitatively assess absolute aggregate counts, the number of EP1536Y and p62 immunoreactive inclusions were counted from different samples corresponding to slides that were all cut at a given neuroanatomic location.

### Quantification of IL6 levels by ELISA and IL-6 immunostaining on brains

Wild type B6/C3H mice received bilateral, intraventricular injections of rAAV-IL-6 on neonatal day P0 and were harvested at 1.5 months. Mice brains were divided sagitally; left hemisphere was snap frozen for biochemical determination of IL-6 levels and right hemisphere was fixed in formalin and paraffin embedded as coronal sections for IL-6 immunohistochemistry. The frozen hemibrains were extracted in RIPA buffer containing 1x Protease inhibitor cocktail (Roche, Indianapolis, IN) and used for biochemical determination of IL-6 protein levels using the Opti EIA mouse IL-6 assay (BD Biosciences) as described earlier [33]. Colorimetric assays were analyzed using the SoftMax data acquisition software (Molecular Devices). For immunohistochemistry, paraffin embedded slides were stained with anti IL-6 antibody (AbCam, 1:200) following antigen retrieval by steaming in Dako Target Retrieval Solution S1699 (modified citrate buffer, pH 6.1). Colorimetric slides were treated with ImmPress reagents (Vector Labs) and visualized with 3, 3’diaminobenzidine followed by hematoxylin counterstaining.

### Statistics and image processing

All statistics were performed using the GraphPad Prism software (GraphPad). Images were mounted using Adobe Photoshop CS4.

## Results

### IL-6 expression induces gliosis in the brains of naïve wild type mice and αSyn fibril injected wild type mice

**Fig. 1:**
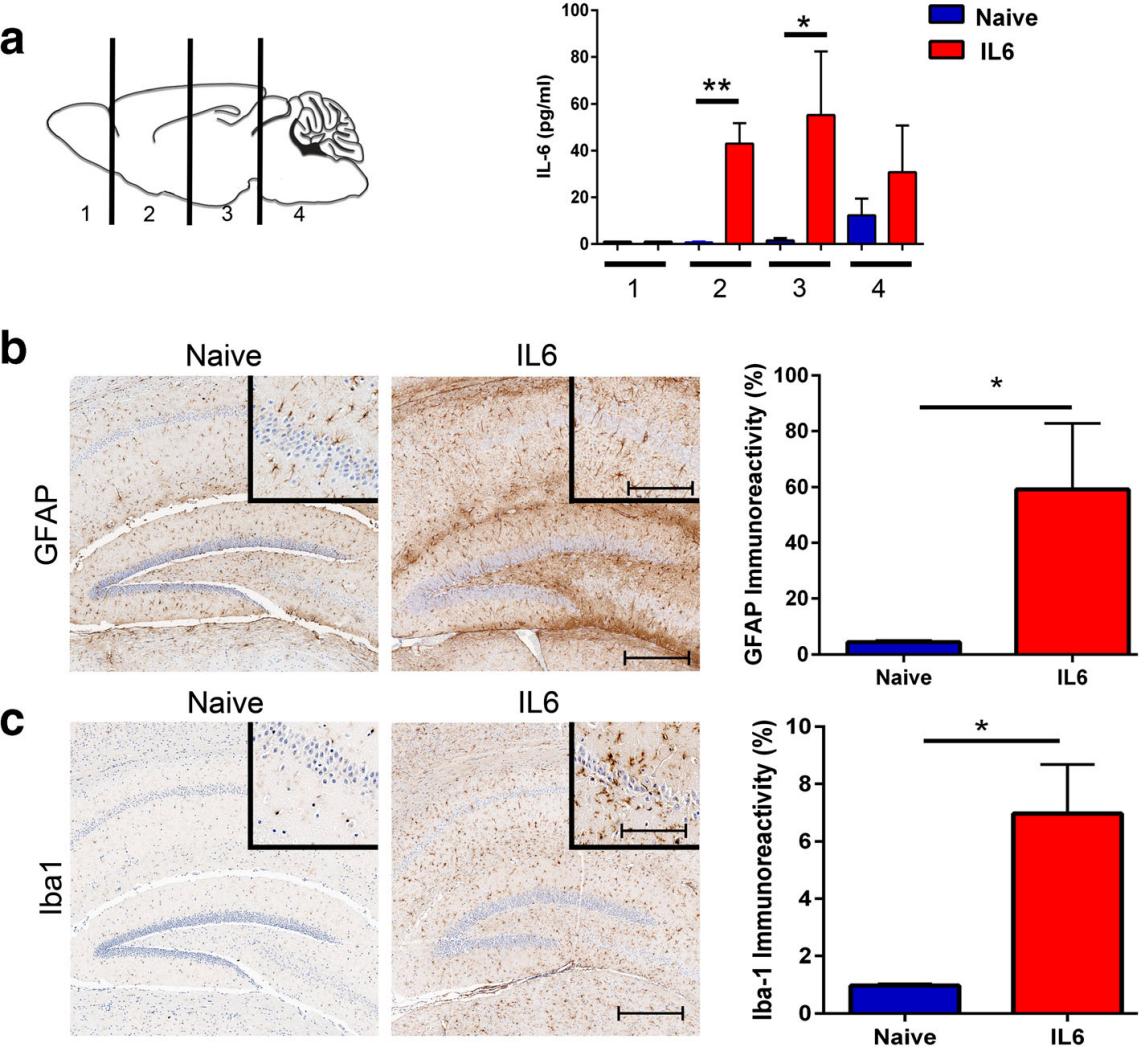
Expression of IL-6 leads to widespread astrogliosis in the forebrain of wild type mice. Wild type mice were injected with rAAV-IL-6 on neonatal day P0 and analyzed at 1.5 months of age for IL-6 protein levels by ELISA (**a**). The numerals (on x- axis) denote the different regions of a sagittally sectioned brain as shown on the model brain on the *left* panel (**a**). Uninjected (naïve) mice were used as controls. Mouse forebrains were also analyzed for astrocyte (GFAP) (**b**) and microglia (Iba-1) activation (**c**). GFAP and Iba-1 immunoreactivity was quantified and depicted across the whole forebrain (**b**–**c**). *n* = 3 mice/group. **p < 0.01, *p < 0.05, Student’s *t* test. Scale bar, 150 μm and 40 μm (inset)

To explore the effect of chronic immune activation in a model of αSyn seeding in wild type mice, neonatal B6/C3H pups were transduced by bilateral injection of rAAV1-Empty vector (rAAV1-EV) or rAAV1-IL-6 into the cerebral ventricles. rAAV1-EV injected animals were used as the control cohort in this study as these rAAV vectors contain the empty pAAV plasmid without any exogenous protein-encoding genes. Mice were aged to 1.5 months and analyzed for the extent of IL-6 expression and astrogliosis (Fig. 1). Direct ELISA determination of IL-6 levels showed that except for the anterior-most (olfactory bulb), IL-6 was upregulated by ~40 fold over control cohorts in forebrain and midbrain areas (Fig. 1a). Immunostaining for IL-6 protein revealed diffuse IL-6 reactivity in the neuropil as well as some cellular staining present throughout the forebrain and midbrain areas compared to control mice (Additional file 1: Figure S1). We also conducted detailed immunohistochemical analysis to assess GFAP immunoreactive astrocytes and Iba-1 reactive microglia in the cortex and hippocampus. We observed widespread astrocytosis and microgliosis in IL-6 overexpressing mice (Fig. 1). Quantification of immunostaining using the positive pixel count program (Aperio ImageScope) showed significant increases in astrocytic staining (↑13.3x in IL-6 mice compared to naïve mice; Fig. 1b) and microglial staining (↑7.1x in IL-6 mice compared to naïve mice; Fig. 1c) in IL-6 expressing mice compared to age-matched naïve mice.

**Fig. 2:**
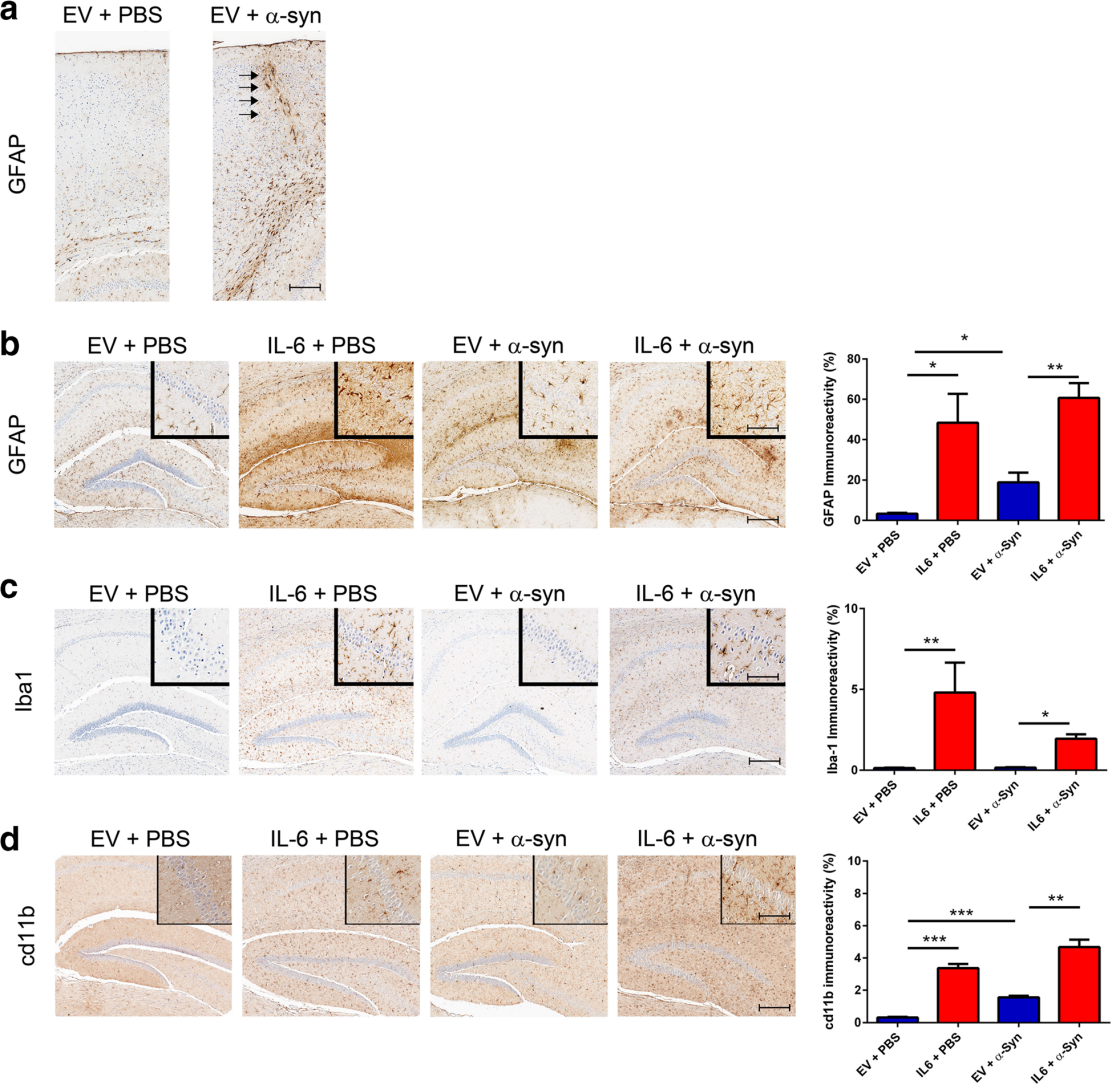
Astroglial activation in wild type mice following intra-hippocampal delivery of αSyn fibrils. Neonatal mice were transduced with rAAV-EV or rAAV-IL-6 and injected with αSyn fibrils or PBS (vehicle) in the hippocampus at 2 months of age. 4 months following the intrahippocampal injections of αSyn fibrils, focal gliosis (GFAP) was observed along the needle tract in rAAV-EV mice (**a**). PBS (vehicle) serves as control (**a**). **b**–**d**. Expression of rAAV-IL-6 alone (IL-6 + PBS) resulted in a significant upregulation of GFAP (*p* = 0.0345), Iba-1 (*p* = 0.0328) and cd11b (*p* = 0.0003) immunoreactivity, compared to EV + PBS cohorts. In the EV cohort, αSyn injection leads to increased GFAP (*p* = 0.0146) and cd11b (*p* = 0.0002) but does not change Iba-1 levels (*p* = 0.2216). In the IL-6 cohort, injection of αSyn leads to a nonsignificant trend in increased GFAP (*p* = 0.2432) and cd11b (*p* = 0.0679) immunoreactivity and causes a non-significant lowering trend in Iba-1 levels (*p* = 0.1002). Whole brain images corresponding to these panels are shown in Additional file 2: Figure S2. *n* = 3–4/cohort. ***p < 0.001, **p < 0.01, *p < 0.05, Student’s *t* test. Scale bar, 150 μm (**a**–**c**) and 40 μm (inset)

Two month old mice transduced with rAAV1-IL-6 or rAAV1-EV were bilaterally injected with 2 μg of wild type mouse αSyn fibrils or sterile saline (PBS) in the hippocampus. Mice were aged to 6 months of age and analyzed for astrogliosis in the hippocampal area. Four different groups were analyzed for astrogliosis: mice injected on neonatal day P0 with rAAV1-EV and subsequently injected in the hippocampus with either PBS or aggregated αSyn and mice injected on neonatal day P0 with rAAV1-IL-6 and subsequently injected in the hippocampus with either PBS or aggregated αSyn (Fig. 2, Additional file 2: Figure S2). Injection of αSyn fibrils in EV cohort induced astrogliosis compared to PBS injected mice (needle track, arrows, Fig. 2a), clearly showing that the presence of exogenous αSyn aggregates activated astrocytes at least focally. Overall, IL-6 + PBS mice show higher induction of astrocytosis (GFAP) and microgliosis (Iba-1 and cd11b) compared to EV + PBS mice (Fig. 2b–d). Though injection of αSyn fibrils also increases cd11b and GFAP immunoreactivity in both IL-6 and EV cohorts (Fig. 2b, d), the extent of astrogliosis and microgliosis induced by αSyn fibrils (between EV + PBS and EV + αSyn cohorts) was considerably lower than the magnitude of glial activation observed in response to IL-6 alone (between IL-6 + PBS and EV + PBS cohorts) (Fig. 2b–d). Surprisingly, there was a non-significant lowering of Iba-1 immunoreactivity in IL-6 group compared to the EV groups following hippocampal delivery of aggregated αSyn (Fig. 2c). We also observed sparse MHCII immunopositive cells specifically around the ventricles in IL-6 alone or αSyn fibril injected cohorts (Additional file 3: Figure S3). MHCII staining was not observed in other areas of the brain, for example hippocampus or cortex (Additional file 3: Figure S3).

### IL-6 expression attenuates αSyn pathology following intrahippocampal injection of preformed αSyn fibrils

**Fig. 3:**
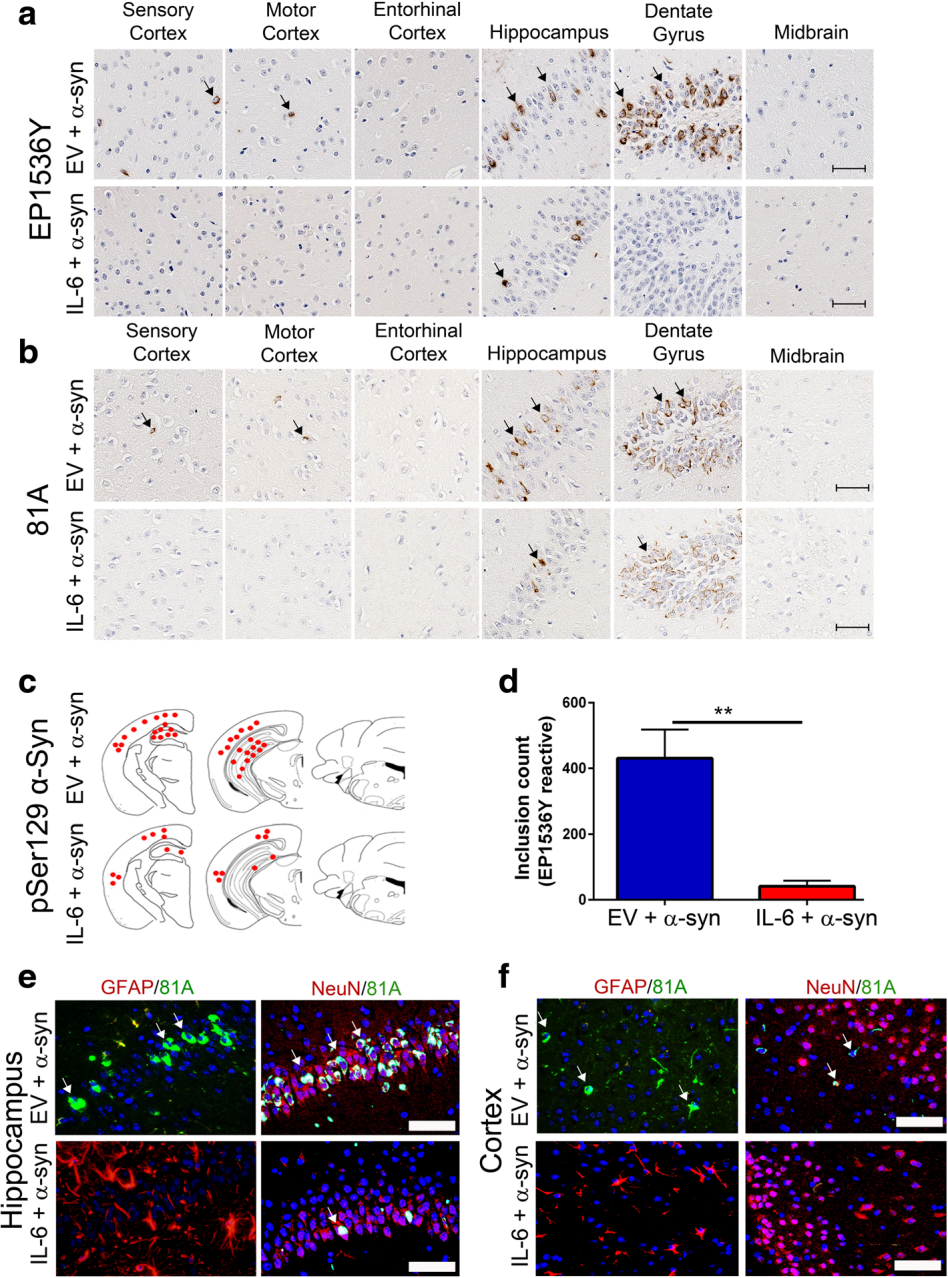
IL-6 reduces αSyn pathology in wild type mice following intra-hippocampal injection of αSyn aggregates. Neonatal wild type mice were injected with rAAV-EV or rAAV-IL-6 and subsequently bilaterally injected in the hippocampus with αSyn fibrils at 2 months of age. Mice were analyzed 4 months post injection with αSyn fibrils in the hippocampus. Representative images of different brain regions stained with two antibodies (EP1536Y and 81A) that recognize the pSer129 epitope on αSyn are shown (**a**–**b**). The pattern and distribution of pSer129 αSyn pathology was similar with both antibodies. αSyn inclusions were mainly found in the hippocampus, dentate gyrus and cortical regions. Mice expressing IL-6 presented with lower levels of αSyn pathology than in EV control cohorts. Hematoxylin was used to counterstain tissues. Scale Bar, 25 μm. *Red dots* depicting rostral/caudal distribution of αSyn inclusions in wild type mice identified by pSer129 immunostaining is presented as a qualitative measure of relative amounts of αSyn burden (**c**). The actual number of αSyn inclusions were counted in EP1536Y stained sections (whole brain) of IL-6 and EV cohorts injected with αSyn aggregates (**d**). **p < 0.01. **e**–**f**, Co-immunofluorescence staining with GFAP (astrocyte marker, Alexa Fluor 594) or NeuN (neuronal marker, Alexa Fluor 594) with 81A antibody (Alexa Fluor 488) shows αSyn inclusions primarily localized in the neurons in the hippocampus (*arrows*, **e**) and cortex (*arrows*, **f**). Overall, intraneuronal αSyn inclusion pathology is reduced in IL-6 expressing mice. *n* = 4–5 mice/cohort. Scale bar, 25 μm

Injections of exogenous αSyn fibrils into predetermined areas of the mouse brain leads to αSyn inclusion pathology in wild type mice [13–15]. Here, we used a similar model of α-synucleinopathy by injecting preformed mouse αSyn fibrils in the hippocampus of 2 month old wild type mice transduced with rAAV1-EV or rAAV1-IL-6 on day P0. 4 months post injection, we detected αSyn pathology throughout the hippocampal CA1-3 and dentate gyrus and to a lesser extent in the sensorimotor cortex (Fig. 3a–c). No αSyn pathology was observed in other regions of the brain. The methodology used in our studies has been standardized in previous publications from our group [7, 13]; since we conducted bilateral seeding and used the whole brain for immunohistochemical assays that allow for accurate quantification, this precluded us from performing any biochemical assays of αSyn. Pathological αSyn was detected using two distinct antibodies raised against phosphorylated Ser129 epitope in αSyn: pSer129/81A [36] and EP1536Y (AbCam). We observed that in IL-6 expressing mice, the induction αSyn pathology was significantly lower in all the areas of the brain examined (Fig. 3a–c). The absolute counts of EP1536Y immunoreactive pSer129-αSyn inclusions in the whole brain showed that there was a 10.4x reduction in IL-6 expressing mice (*p* = 0.0023) (Fig. 3d). Using immunofluorescence colocalization in the hippocampus (Fig. 3e) and cortex (Fig. 3f), we confirmed that perikaryal pSer129/81A inclusions were mostly present in neurons (arrows, NeuN panel) in response to exogenous αSyn fibrils in EV cohorts. Intraneuronal αSyn inclusion staining was dramatically reduced in the hippocampus and cortex of IL-6 expressing mice compared to the EV cohort (Fig. 3e–f).

### p62-reactive pathology induced by αSyn fibrils is attenuated in IL-6 expressing mice

**Fig. 4:**
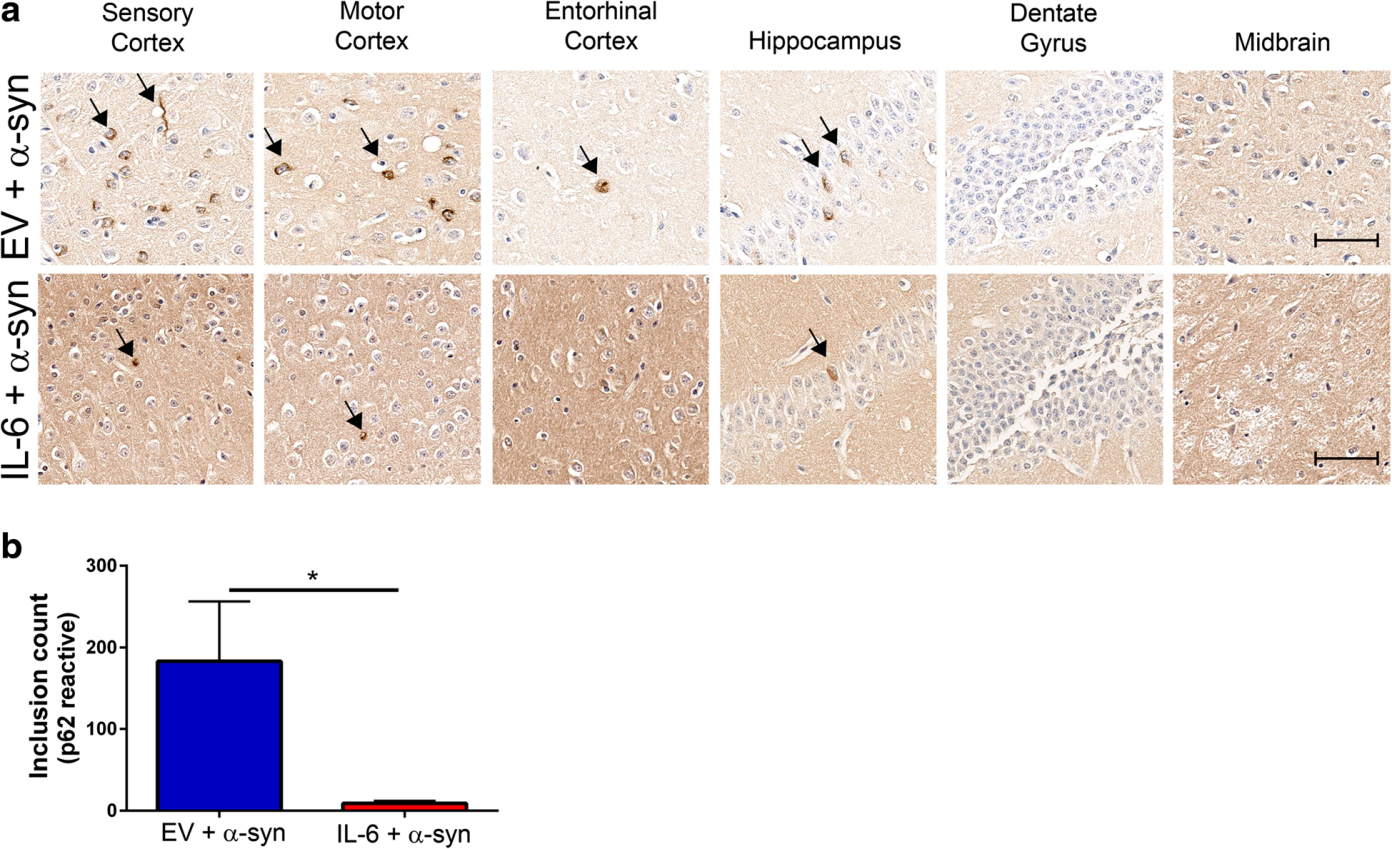
p62 immunoreactive inclusion pathology is reduced in IL-6 expressing mice injected in the hippocampus with αSyn fibrils. Neonatal wild type mice were injected with rAAV-EV or rAAV-IL-6 and subsequently bilaterally injected in the hippocampus with αSyn fibrils at 2 months of age. Mice were analyzed 4 months post injection with αSyn fibrils in the hippocampus. Representative p62 staining is shown in different brain regions of rAAV-EV or rAAV-IL-6 mice following intra-hippocampal injection with αSyn fibrils (*arrows*, **a**). IL-6 expressing mice show considerably lower p62 levels than EV cohorts in response to hippocampal αSyn challenge. The number of hippocampal and cortical p62 inclusions were counted in IL-6 and EV cohorts injected with αSyn aggregates (**b**). *p < 0.05. *n* = 4–5 mice/cohort. Scale bar, 25 μm

The extent of αSyn pathology in EV or IL-6 expressing wild type mice was also confirmed with anti-p62/sequestosome antibody, which is a component of Lewy body inclusions [38, 39]. Perikaryal and cytoplasmic p62 staining was localized in the cortex and hippocampus of EV injected mice, while IL-6 expressing mice had considerably lower amounts of p62 stained inclusions in both areas (Fig. 4a, arrows). Overall, there was a 95% decrease in p62-reactive cell body count in the hippocampus and cortex (*p* < 0.05) (Fig. 4b). We further confirmed that age-matched naïve mice (EV and IL-6 cohorts) which were not challenged with αSyn fibrils did not show accumulation of p62 inclusions (Additional file 4: Figure S4), confirming that the p62 induction is directly associated with the extent of pathological αSyn inclusions in these mice, and not with inflammatory activation.

### IL-6 induced inflammatory milieu also limits the induction of argyrophilic inclusions by αSyn fibrils

**Fig. 5:**
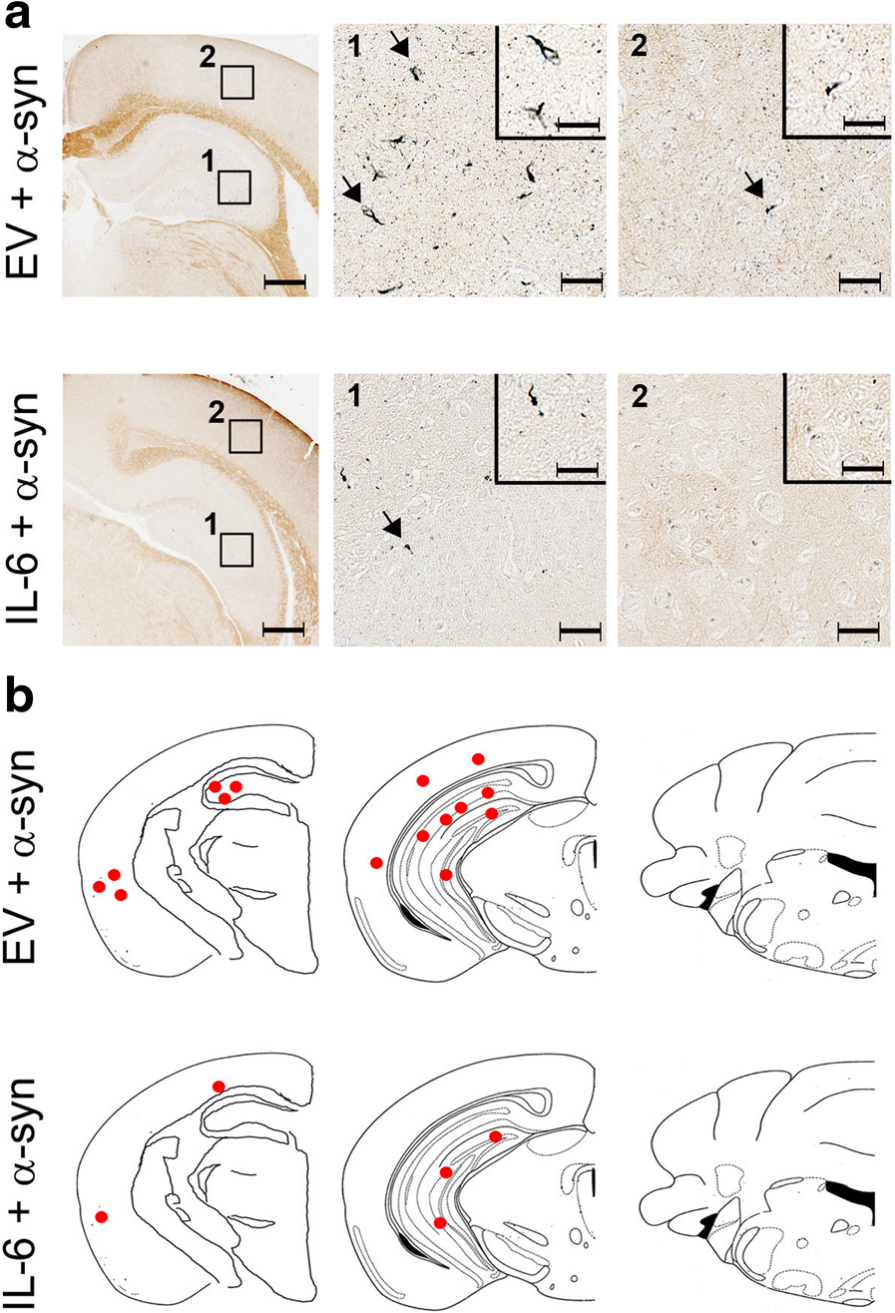
Argyrophilic inclusion pathology is attenuated in IL-6 expressing mice injected in the hippocampus with αSyn fibrils. Neonatal wild type mice were injected with rAAV-EV or rAAV-IL-6 and subsequently bilaterally injected in the hippocampus with αSyn fibrils at 2 months of age. Mice were analyzed 4 months post injection with αSyn fibrils in the hippocampus. Representative images of hippocampal and cortical regions of mice stained with modified Campbell-Switzer silver staining show that mice overexpressing IL-6 have fewer silver-positive inclusions than control (EV) mice (**a**). Red dots show the comparative distribution of argyrophilic inclusions along rostral-caudal axis as identified by modified Campbell-Switzer silver staining (**b**). *n* = 4–5 mice/cohort. Scale bar, 150 μm (*left panel*) and for middle and right panels, 30 μm and 15 μm (inset)

Using Campbell-Switzer silver staining which detects Lewy body pathology [40], we examined the patterns of cytoplasmic inclusion pathology in EV and IL-6 cohorts following intrahippocampal delivery of αSyn fibrils. Consistent with patterns of pSer129 αSyn immunopositivity, we observed argyrophilic inclusions only in the brain regions contiguous to the injection site. In the EV cohorts, there was copious perikaryal staining mostly in the hippocampus and occasional staining was noted in the cortex. In the IL-6 expressing mice, the intracellular silver staining was considerably lower in both the hippocampus and cortex (Fig. 5a–b).

### IL-6 induced attenuation of αSyn pathology is an early event

**Fig. 6:**
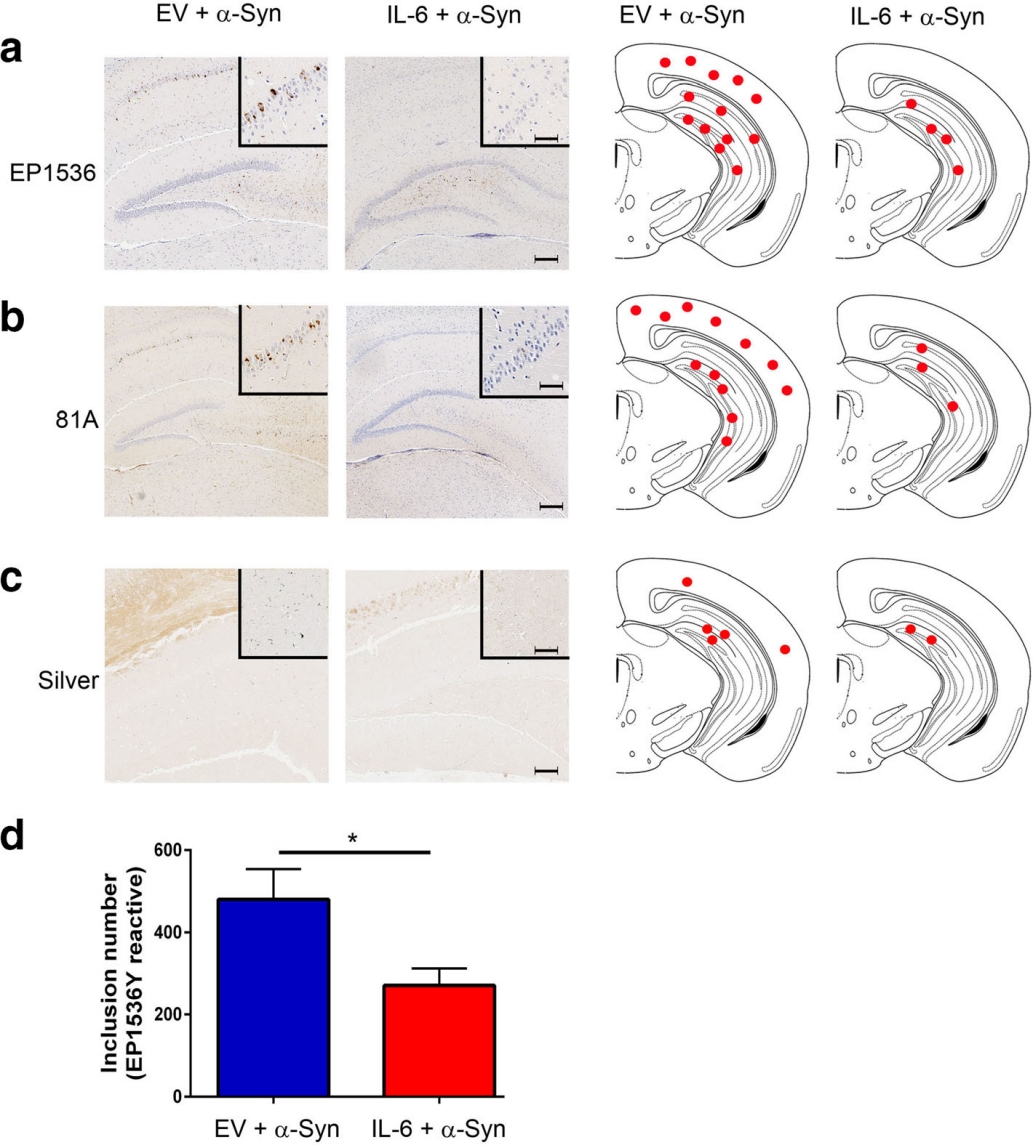
Reduced αSyn pathology in IL-6 expressing mice at 2 months following intra-hippocampal injection of αSyn fibrils. Neonatal wild type mice were injected with rAAV-EV or rAAV-IL-6 and subsequently bilaterally injected in the hippocampus with αSyn fibrils at 2 months of age. pSer129 αSyn immunoreactive pathology was assessed in wild type mice 2 months post intra-hippocampal αSyn challenge. Immunolabeling with EP1536Y and 81A antibodies demonstrate reduced pathological αSyn levels in IL-6 mouse brains (**a**–**b**). Argyrophilic inclusions were also reduced in IL-6 cohort compared to the EV cohort (**c**). Accompanying panels on the right display the comparative distribution of pSer129 immunoreactive αSyn pathology on a representative brain map. The number of αSyn inclusions were counted in EP1536Y stained sections (whole brain) of IL-6 and EV cohorts injected with αSyn aggregates (**d**). *p < 0.05. *n* = 4–5 mice/cohort. Scale bar, 300 μm and 80 μm (inset)

To further investigate the timeline of the effect observed in IL-6 expressing mice, we assessed a second cohort of mice 2 months following intrahippocampal challenge with αSyn fibrils. Similar to earlier experiments, neonatal B6/C3H pups were transduced by bilateral injection of rAAV2/1-EV or rAAV2/1-IL-6 into the cerebral ventricles, aged to 2 months and then injected with αSyn fibrils in the hippocampus. When these mice were analyzed 2 months post injection, both GFAP and Iba-1 astrogliosis were upregulated in the forebrain areas in IL-6 expressing mice compared to EV cohorts (Additional file 5: Figure S5A–B). Examination of pSer129 αSyn immunopositivity in this cohort (2 months αSyn challenge) revealed a similar pattern that was observed in the first cohort (4 months αSyn challenge): IL-6 expressing mice had lower αSyn burden compared to EV cohorts when examined 2 months post α-Syn challenge (Fig. 6a–d). The absolute counts of EP1536Y immunoreactive pSer129-α-Syn inclusions in the whole brain showed that there was a 1.7x reduction in IL-6 expressing mice (*p* = 0.0251) (Fig. 6d). In IL-6 cohorts, αSyn immunopositivity was mostly restricted to the hippocampus whereas in the EV cohorts, perikaryal αSyn staining was observed in both hippocampus and cortex. There was a corresponding decrease in argyrophilic inclusions in the IL-6 injected mice compared to the EV injected mice (Fig. 6c).

## Discussion

Here we have investigated the effect of inflammatory immune activation on the induction of αSyn pathology in wild type mice following intra-hippocampal delivery of pre-fibrillized αSyn aggregates. We show that expression of IL-6 in two independent time progressive cohorts of wild type mice results in 1) widespread and massive astrogliosis, 2) attenuation of induced αSyn pathology in mice challenged with exogenous αSyn aggregates and 3) concurrent reduction in p62 and argyrophilic inclusion pathology in mice injected with αSyn. Our study is in agreement with earlier reports that αSyn can be readily endocytosed by astrocytes [16] and this is potentially an endogenous mechanism for rebalancing proteostasis abnormalities via lysosomal degradation [17]. Taken together, this suggests that prior activation of astroglia alters the innate immune milieu to generate a beneficial response in wild type mouse brains challenged with exogenous αSyn aggregates. However, whether IL-6 induced inflammatory activation will have a different outcome in a chronic setting, especially with regard to neurodegeneration and behavioral outcome measures, remains to be investigated.

Templated conformational alterations in intracellular αSyn and its subsequent secretion across neuroanatomic junctions has emerged as a possible mechanism of disease progression in α-synucleinopathies [4]. However, little is known about the non-cell autonomous effects of extracellular αSyn on innate immunity in the CNS. More importantly, whether innate immune based strategies could potentially induce rapid uptake and degradation of extracellular αSyn in vivo by immune cells has not been studied. While chronic inflammation can have a detrimental role in CNS homeostasis [41], whether transient activation of astrocytes and microglia can be harnessed as a disease modifying therapy in αSyn models of PD is still unknown.

There are multiple reports showing that various therapies can attenuate αSyn pathology in transgenic mouse models (reviewed in [42]). However, only one previous report, using a peripherally administered monoclonal anti-αSyn antibody showed that prefibrillar αSyn aggregate induced α-synucleinopathy can be blocked in vivo [43]. Additionally, the reported safety and preliminary efficacy of the ongoing AFFITOPE® PD01A (AFFiRis AG) trials in human patients lends support to the idea that activating the patient’s immune system to generate anti-αSyn response may be potentially beneficial in limiting the progression of αSyn pathology. Herein, we show that pro-inflammatory preconditioning significantly attenuates induction of endogenous αSyn pathology in wild type mice. Further, our data seems to suggest that in addition to potential immune scavenging of exogenous αSyn aggregates around the time of administration, IL-6 induced gliosis was instrumental in removing the secondary pathological forms of αSyn at a later timepoint. The mechanism of such time-progressive reduction remains unknown but given the present understanding, it is likely that attenuation of αSyn pathology may occur via upregulation of phago-lysosomal function of the glial cells (reviewed in [44]). Both the autophagy-lysosomal pathway and the ubiquitin proteasome system have been shown to play crucial roles in αSyn clearance [45]. However, there may well be additional clearance mechanisms underlying the reduction in αSyn pathology observed in our study. Our data thus provides a second and unique instance where manipulating the immune system provides a beneficial response in a model of exogenous fibril-induced αSyn pathology. There are some caveats to such immune manipulations. Given that microarray expression data suggest that aging itself skews the human brain towards a pro-inflammatory state, it is interesting to consider how those age-induced alterations might modify the spread of neurodegenerative pathology in humans [46]. In particular, special cognizance ought to be placed on harnessing the beneficial nature of innate immune function vis-a-vis the potentially harmful outcomes of a chronic response.

Our present data as well as earlier reports show that CNS injection of exogenous αSyn aggregates leads to focal gliosis, especially discernible along the needle track [7]. Exogenous αSyn aggregates may cause glial dysfunction followed by intracellular proteostasis resembling neuropathological changes in synucleinopathies [7, 9]. On the other hand, astroglial activation may provide trophic support to the CNS in rodent models of PD and therefore may have beneficial neuroprotective effects overall [47, 48]. A major function of glial cells is to scavenge debris [49] and it is possible that facilitating clearance of extracellular αSyn may attenuate inter-neuronal transfer of αSyn and support normal homeostasis. Interestingly, αSyn is thought to be taken up by astrocytes around axon terminals in brains of PD patients [50], which was demonstrated in cell culture using exogenous αSyn [16]. Both astrocytes and microglia can internalize and degrade extracellular and cell-derived αSyn [11, 17, 29, 30, 51, 52]. Two microglial receptors, TLR2 and TLR4, have been identified as possible endogenous receptors responsible for αSyn internalization; indeed, TLR4 ablation reduces αSyn clearance and exacerbates neurodegeneration [30]. Therefore, such astrocytic and microglial scavenging and clearance mechanisms hold promise in preventing the spread of αSyn pathology during inter-cellular transfer of αSyn seeds.

Although the general dogma in many neurodegenerative disorders is that inflammatory stimuli might promote disease [53], data in preclinical models of Alzheimer’s disease demonstrate that activating the immune system can attenuate the underlying proteinopathy [33, 54–57]. We and others have demonstrated that at least early in the disease process, inflammatory activation in the CNS can have positive disease modifying effects in mouse models of Alzheimer’s disease [33, 54, 58, 59]; similarly, in this study, we demonstrate that at least in an acute scenario, IL-6 induced innate immune activation can have a protective outcome in wild type mice following exogenous αSyn challenge. To our knowledge the current data presented here is the first to show that an inflammatory interventional strategy may also work in a mouse model of seeded synucleinopathy. However, in Lewy Body diseases, the role of individual cytokines still remains to be elucidated (reviewed in [60]). Different inflammatory cytokines have been shown to be correlated with the disease process in mouse models of nigro-striatal degeneration as well as in Parkinsonian patients. Both IFN-γ and TNFα have been shown to be associated with neurodegeneration in PD mouse models [61–63] while the role of other cytokines, such as IL-6 and IL-4 that are upregulated in PD patients [64], have not been studied before in mouse models of synucleinopathies. In particular, whether any of these latter cytokine signaling pathways can modify induction and progression of α-synucleinopathy and therefore have translatable disease modifying outcomes is of paramount interest in therapeutics against synucleinopathies.

## Conclusion

Here, we show that inflammatory priming of the CNS using IL-6 attenuates αSyn inclusion pathology in wild type mice. Our data adds novel insights into harnessing innate immunity as disease modifying therapies in synucleinopathies and further identifies potential immune pathways for therapeutic intervention in these diseases.

## Additional files

Additional file 1: Figure S1. Depiction of IL-6 staining in AAV injected mice. Mice were injected at neonatal day P0 with rAAV-IL-6 and analyzed at 1.5 months of age. Representative whole brain images of naïve and IL-expressing mouse brain sections stained with IL-6 are presented. Magnified images from selected areas of the brain (numbered) are presented in the bottom panels. Scale bar, 600 μm (top) and 15 μm (bottom panels). (185 kb)
Additional file 2: Figure S2. Depiction of inflammatory activation in IL-6 expressing mice injected with αSyn fibrils. Mice were injected at neonatal day P0 with rAAV-IL-6 or rAAV-EV and subsequently injected in the hippocampus with αSyn at 2 months of age. Mice were analyzed 4 months post injection with αSyn fibrils in the hippocampus. Whole brain images of Empty vector (EV) and IL-6 expressing mouse brain sections that were injected with αSyn fibrils or PBS in the hippocampus are shown. Representative images stained with GFAP, Iba-1 and cd11b. These images correspond to the high power magnified images shown in Fig. 2b–d. Scale bar, 600 μm. (4.74 MB)
Additional file 3: Figure S3. MHCII staining in IL-6 expressing mice injected with αSyn fibrils or PBS. Mice were injected at neonatal day P0 with rAAV-IL-6 or rAAV-EV and subsequently injected in the hippocampus with αSyn at 2 months of age. Mice were analyzed 4 months post injection with αSyn fibrils in the hippocampus. Representative sections stained with MHCII antibody show that CNS expression of IL-6 alone as well as injection of αSyn fibrils leads to MHCII immunoreactivity around the ventricles. Other regions of the brain, including the hippocampus shown here, do not show any detectable MHCII immunostaining. Scale bar, 80 μm. (2.73 MB)
Additional file 4: Figure S4. IL-6 expression by itself does not alter p62 levels. Mice were injected at neonatal day P0 with rAAV-IL-6 or rAAV-EV and subsequently injected in the hippocampus with αSyn at 2 months of age. Mice were analyzed 4 months post injection with αSyn fibrils in the hippocampus. Representative sections stained with anti-p62 antibody shows that CNS expression of IL-6 by itself does not produce any overt changes in p62 expression. Scale bar, 80 μm. (1.49 MB)
Additional file 5: Figure S5. Astrocytosis and microgliosis in IL-6 expressing mice injected with αSyn. Mice were injected at neonatal day P0 with rAAV-IL-6 or rAAV-EV and subsequently injected in the hippocampus with αSyn at 2 months of age. Mice were analyzed 2 months post hippocampal injection. Presence of IL-6 increases both GFAP and Iba-1 immunoreactivity (A-B) compared to EV cohort. Red dots depicting distribution of glial immunoreactivity in and around the αSyn injection site are presented on the right hand panel in a brain schematic. *n* = 5 mice/cohort. Scale bar, 150 μm and 40 μm (inset). (1.18 MB)

## Abbreviations

CNS: Central nervous system
DAMP: Damage associated molecular patterns
DLB: Dementia with Lewy bodies
EV: Empty AAV vector containing no expression cassette
GFAP: Glial fibrillary acidic protein
Iba-1: Ionized calcium-binding adapter molecule 1
IL-6: Interleukin-6
MSA: Multiple system atrophy
PBS: Phosphate buffered saline
PD: Parkinson’s disease
rAAV1: Recombinant adeno associated virus capsid 1
αSyn: α-synuclein
