# Supplementary figures and images for: Inflammatory pre-conditioning restricts the seeded induction of α-synuclein pathology in wild type mice
Source: Mol Neurodegener. 2017 Jan 3;12:1. doi: 10.1186/s13024-016-0142-z (PMC5210310; doi:10.1186/s13024-016-0142-z)

Naive

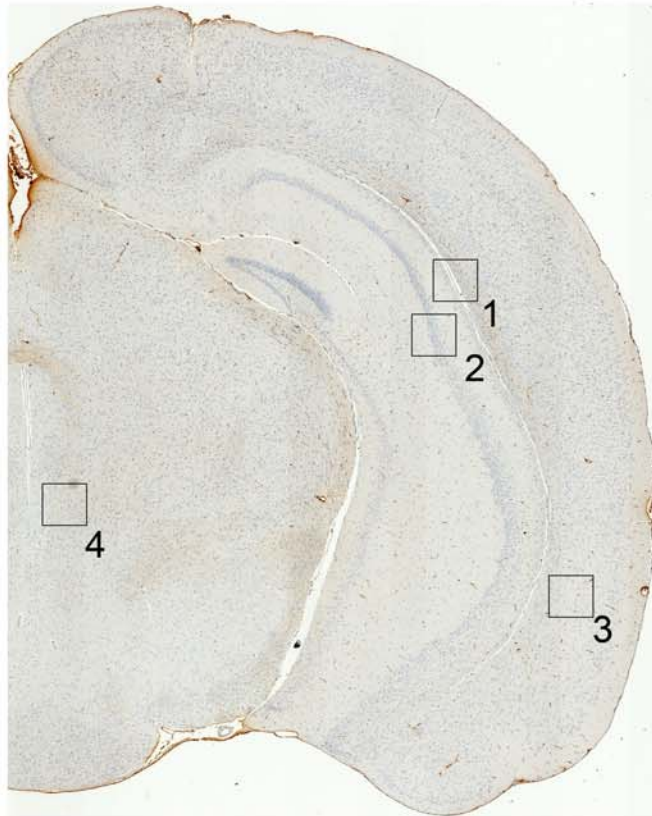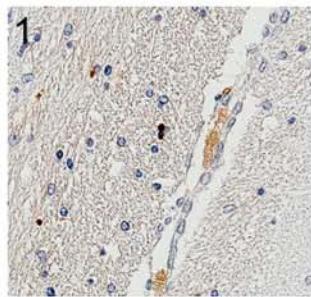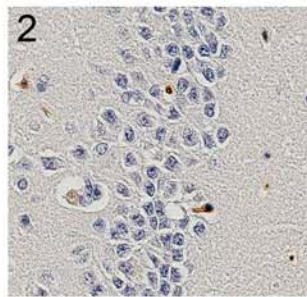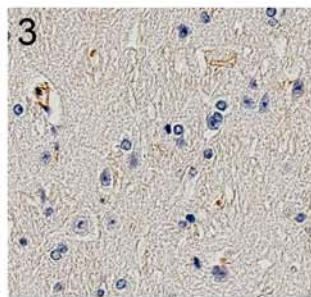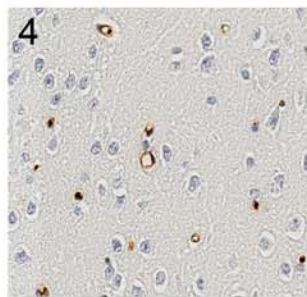

IL-6

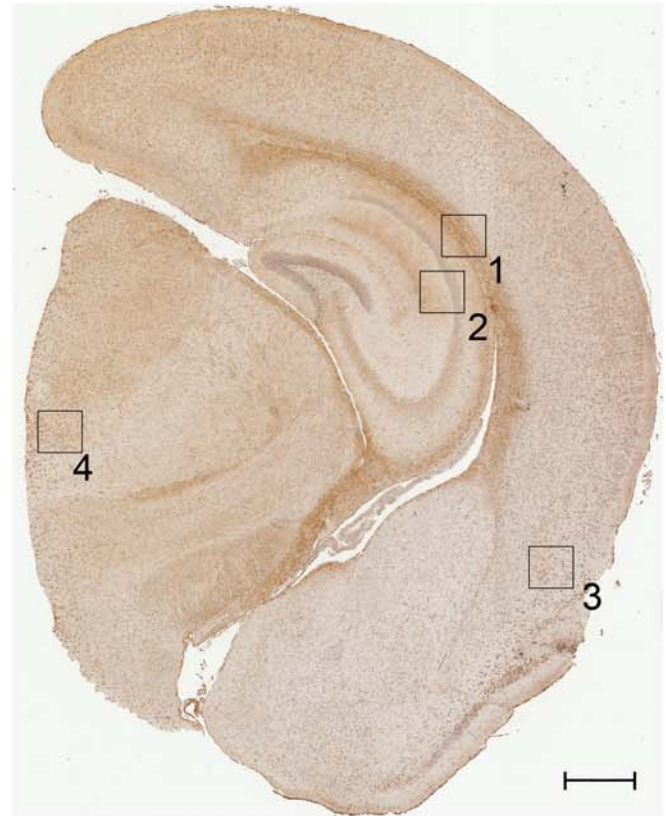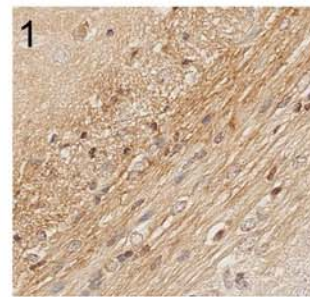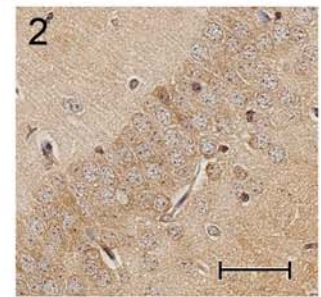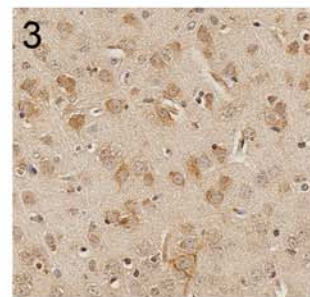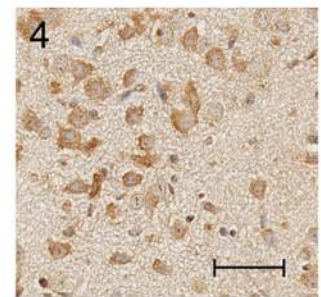

Figure S1

Supplement: Additional file 1: Figure S1. — Depiction of IL-6 staining in AAV injected mice. Mice were injected at neonatal day P0 with rAAV-IL-6 and analyzed at 1.5 months of age. Representative whole brain images of naïve and IL-expressing mouse brain sections stained with IL-6 are presented. Magnified images from selected areas of the brain (numbered) are presented in the bottom panels. Scale bar, 600 μm (top) and 15 μm (bottom panels). (185 kb) [file 13024_2016_142_MOESM1_ESM.pdf]

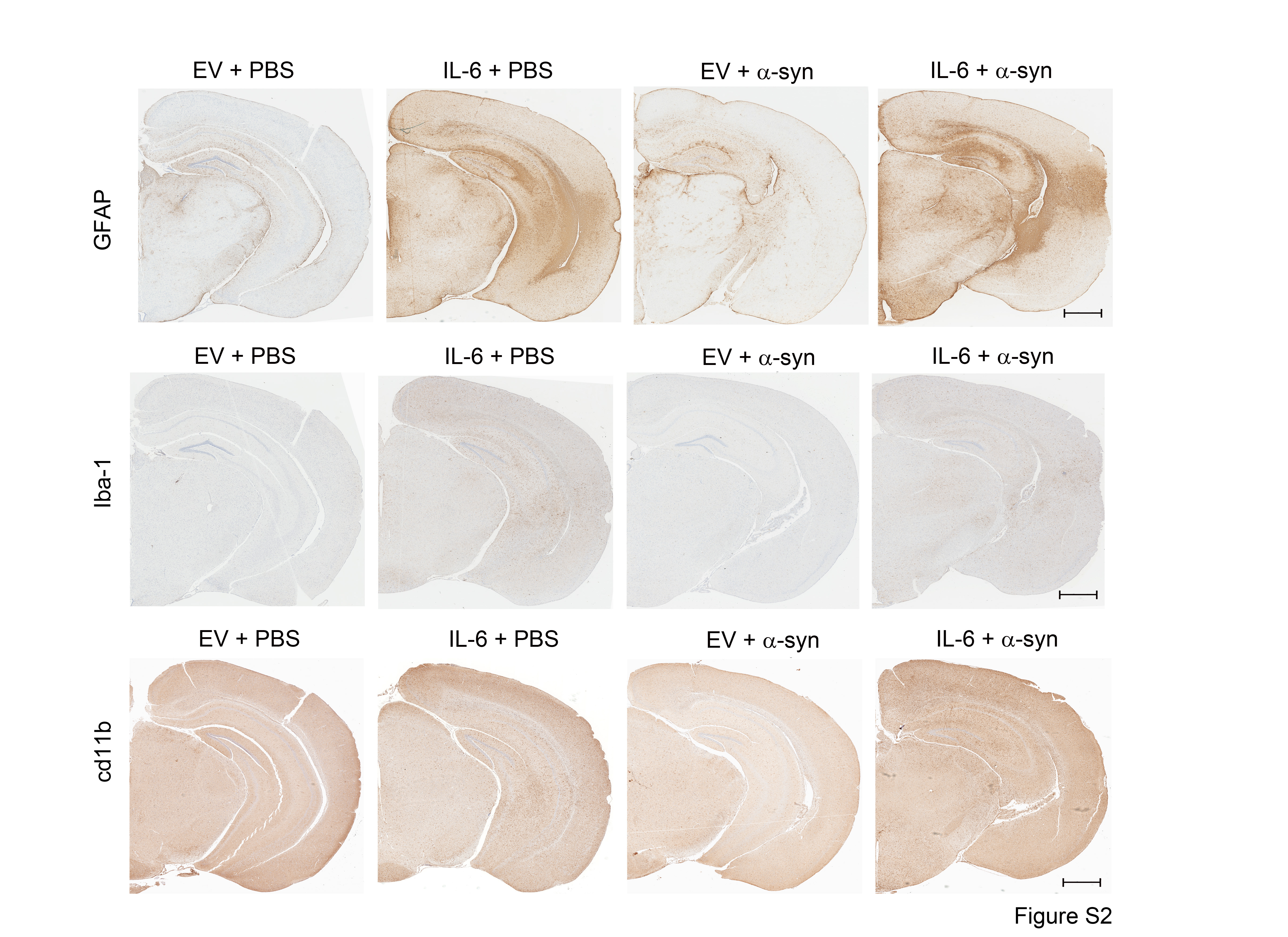

Supplement: Additional file 2: Figure S2. — Depiction of inflammatory activation in IL-6 expressing mice injected with αSyn fibrils. Mice were injected at neonatal day P0 with rAAV-IL-6 or rAAV-EV and subsequently injected in the hippocampus with αSyn at 2 months of age. Mice were analyzed 4 months post injection with αSyn fibrils in the hippocampus. Whole brain images of Empty vector (EV) and IL-6 expressing mouse brain sections that were injected with αSyn fibrils or PBS in the hippocampus are shown. Representative images stained with GFAP, Iba-1 and cd11b. These images correspond to the high power magnified images shown in Fig. 2b–d. Scale bar, 600 μm. (4.74 MB) [file 13024_2016_142_MOESM2_ESM.jpg]

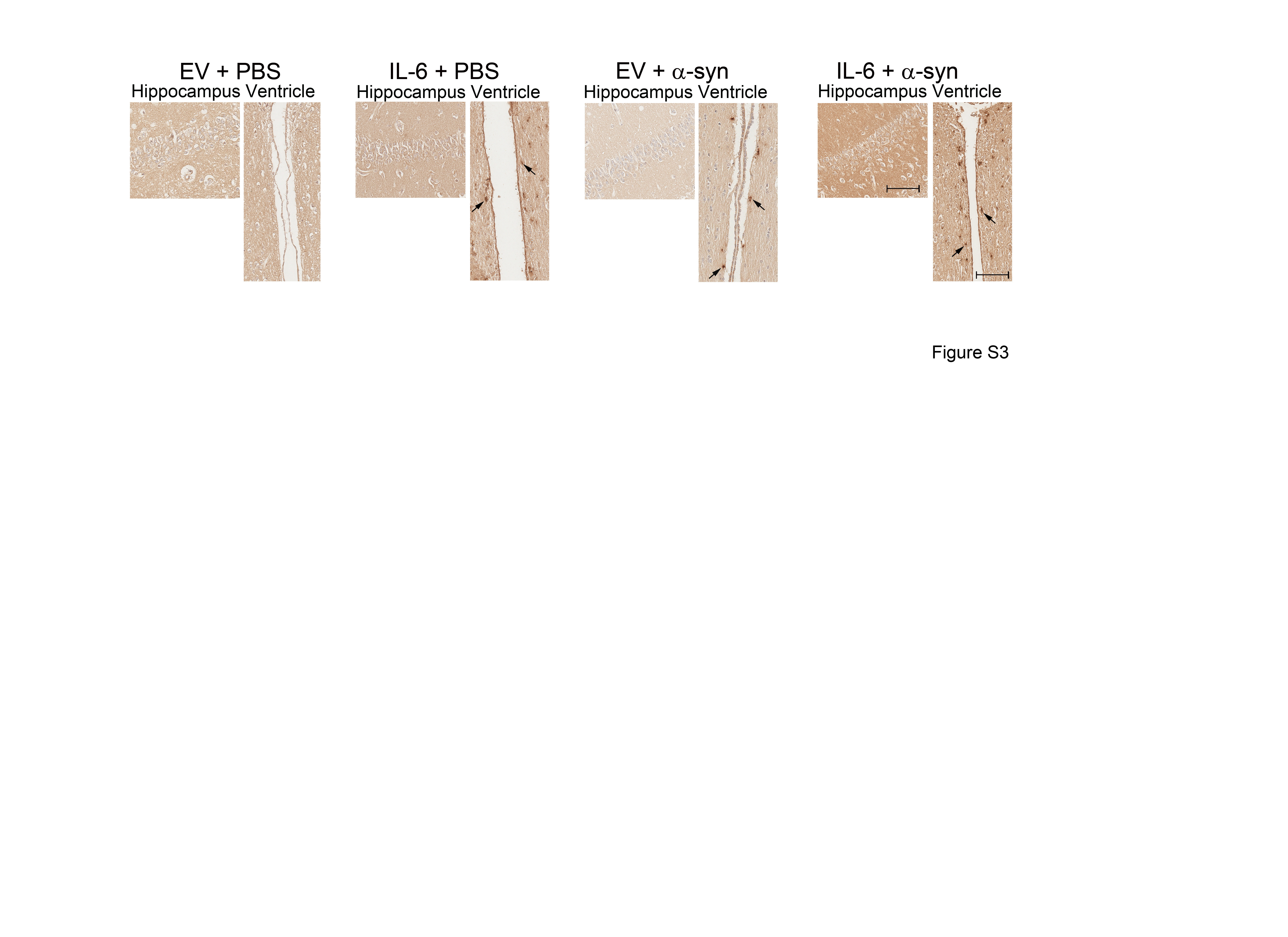

Supplement: Additional file 3: Figure S3. — MHCII staining in IL-6 expressing mice injected with αSyn fibrils or PBS. Mice were injected at neonatal day P0 with rAAV-IL-6 or rAAV-EV and subsequently injected in the hippocampus with αSyn at 2 months of age. Mice were analyzed 4 months post injection with αSyn fibrils in the hippocampus. Representative sections stained with MHCII antibody show that CNS expression of IL-6 alone as well as injection of αSyn fibrils leads to MHCII immunoreactivity around the ventricles. Other regions of the brain, including the hippocampus shown here, do not show any detectable MHCII immunostaining. Scale bar, 80 μm. (2.73 MB) [file 13024_2016_142_MOESM3_ESM.jpg]

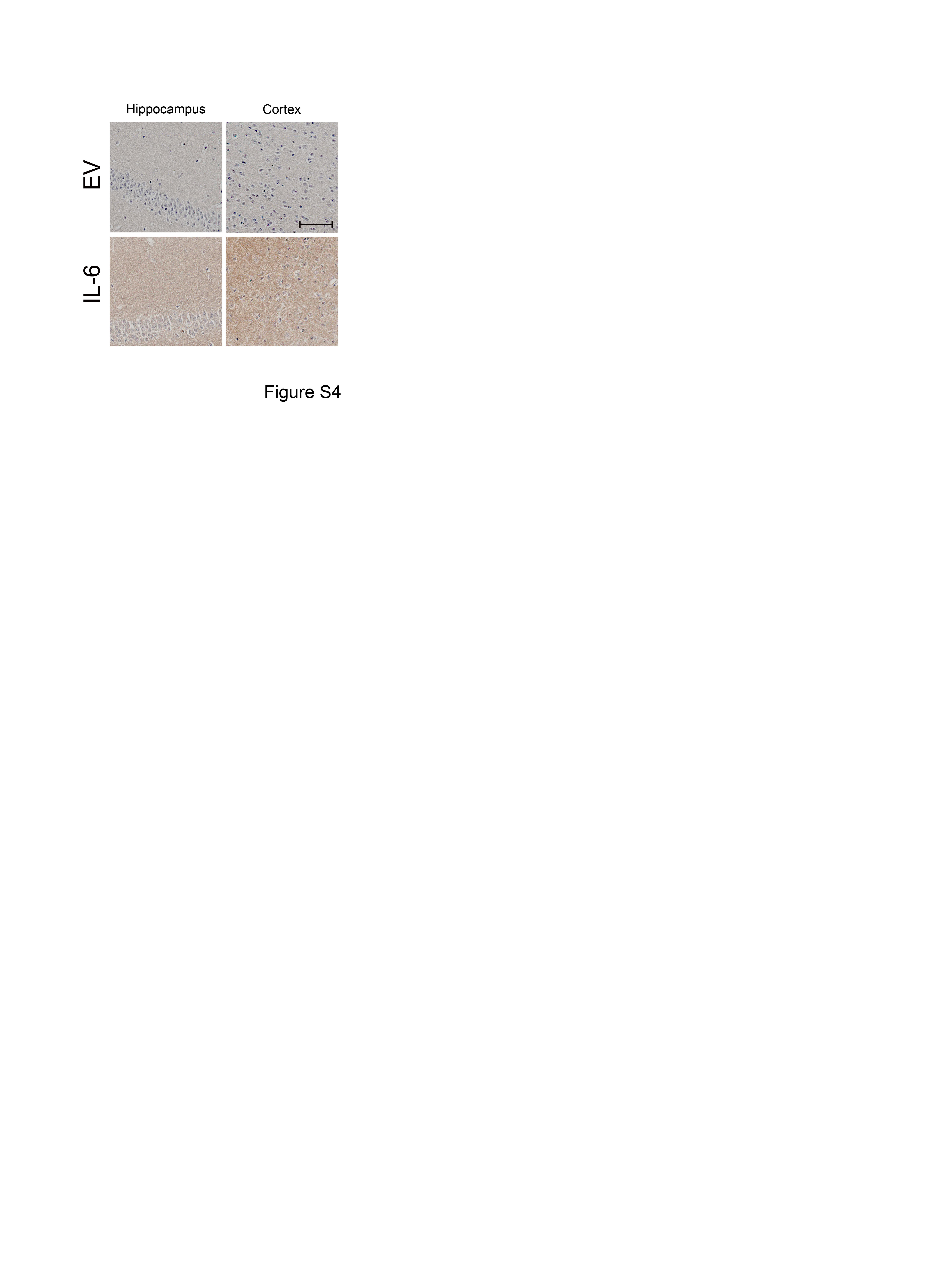

Supplement: Additional file 4: Figure S4. — IL-6 expression by itself does not alter p62 levels. Mice were injected at neonatal day P0 with rAAV-IL-6 or rAAV-EV and subsequently injected in the hippocampus with αSyn at 2 months of age. Mice were analyzed 4 months post injection with αSyn fibrils in the hippocampus. Representative sections stained with anti-p62 antibody shows that CNS expression of IL-6 by itself does not produce any overt changes in p62 expression. Scale bar, 80 μm. (1.49 MB) [file 13024_2016_142_MOESM4_ESM.jpg]

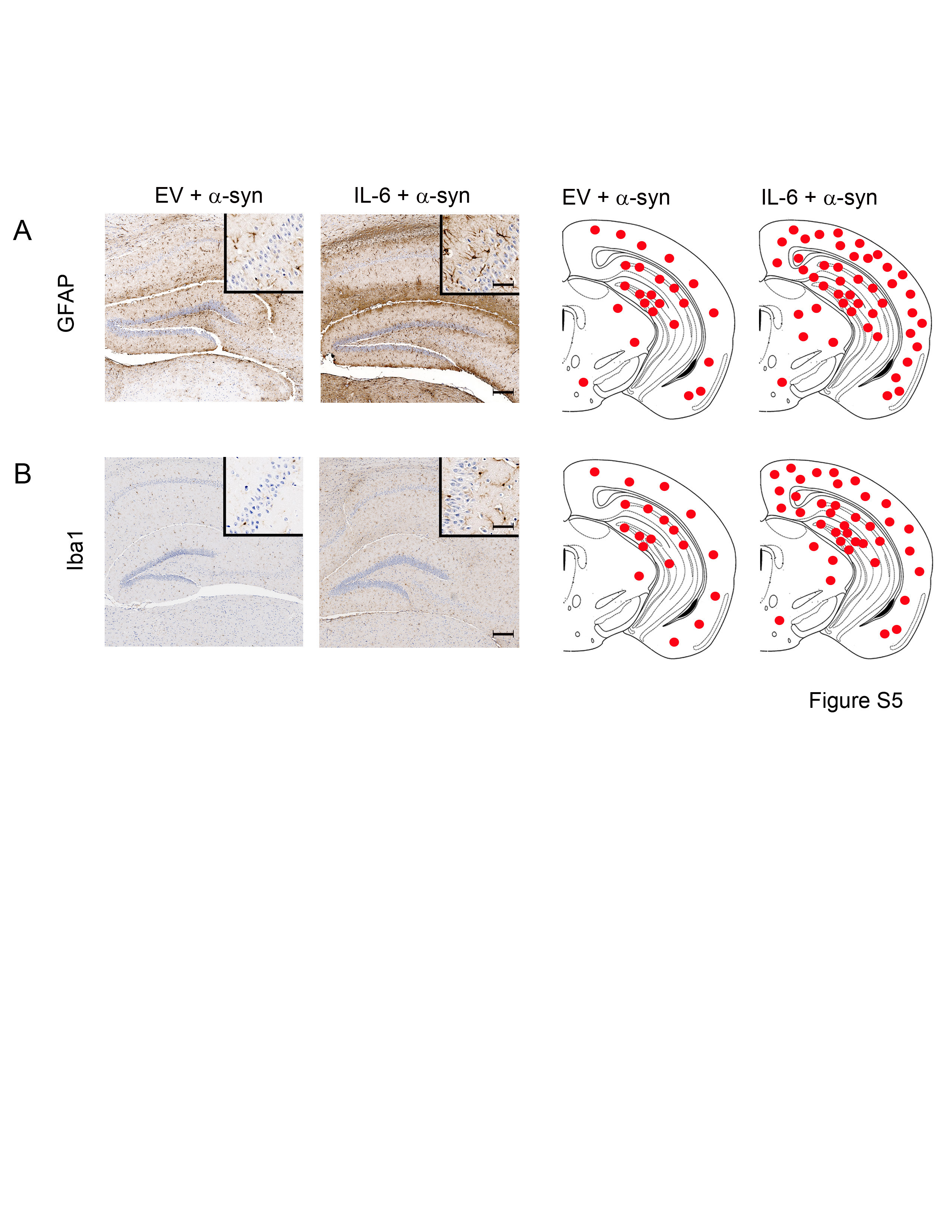

Supplement: Additional file 5: Figure S5. — Astrocytosis and microgliosis in IL-6 expressing mice injected with αSyn. Mice were injected at neonatal day P0 with rAAV-IL-6 or rAAV-EV and subsequently injected in the hippocampus with αSyn at 2 months of age. Mice were analyzed 2 months post hippocampal injection. Presence of IL-6 increases both GFAP and Iba-1 immunoreactivity (A-B) compared to EV cohort. Red dots depicting distribution of glial immunoreactivity in and around the αSyn injection site are presented on the right hand panel in a brain schematic. n = 5 mice/cohort. Scale bar, 150 μm and 40 μm (inset). (1.18 MB) [file 13024_2016_142_MOESM5_ESM.jpg]
